# Magnetically Assisted Immobilization-Free Detection of microRNAs Based on the Signal Amplification of Duplex-Specific Nuclease

**DOI:** 10.3390/bios13070699

**Published:** 2023-06-30

**Authors:** Gang Liu, Ming La, Jiwei Wang, Jiawen Liu, Yongjun Han, Lin Liu

**Affiliations:** 1 College of Chemistry and Chemical Engineering, Anyang Normal University, Anyang 455000, China; 2 School of Chemistry and Environmental Engineering, Pingdingshan University, Pingdingshan 467000, China

**Keywords:** microRNAs, double specific nuclease, magnetic bead, immobilization-free, homogeneous analysis

## Abstract

The double specific nuclease (DSN)-based methods for microRNAs (miRNAs) detection usually require the immobilization of DNA probes on a solid surface. However, such strategies have the drawbacks of low hybridization and cleavage efficiency caused by steric hindrance effect and high salt concentration on the solid surface. Herein, we proposed an immobilization-free method for miRNA detection on the basic of DSN-assisted signal amplification. The biotin- and fluorophore-labeled probes were captured by streptavidin-modified magnetic beads through streptavidin–biotin interactions, thus producing a poor fluorescence signal. Once the DNA probes were hybridized with target miRNA in solution to form DNA-miRNA duplexes, DNA stands in the duplexes would be selectively digested by DSN. The released target miRNA could initiate the next hybridization/cleavage recycling in the homogeneous solution, finally resulting in the release of numerous fluorophore-labeled fragments. The released fluorophores remained in solution and emitted strong fluorescence after treatment by the streptavidin-modified magnetic beads. The immobilization-free method achieved the assays of miRNA-21 with a detection limit down to 0.01 pM. It was employed to evaluate the expression levels of miRNA-21 in different cancer cells with satisfactory results.

## 1. Introduction

MicroRNAs (miRNAs) are a group of endogenous, non-coding RNAs that play an important role in physiological and pathological processes. The abnormal expression levels of miRNAs are associated with many human diseases, including cancers and degenerative diseases [1,2]. Therefore, miRNAs have been regarded as the credible biomarkers for early diagnosis and treatment evaluation of diseases. Reverse transcription real-time quantitative polymerase chain reaction (RT-qPCR) is the currently used gold standard method for monitoring the expression levels of miRNAs. The method shows high specificity and sensitivity but requires complex operation procedures, specialized equipment, and a clean experimental environment [3]. Thus, it is meaningful and highly desired to develop a simple and sensitive detection system for the real-time monitoring of the level of miRNAs.

As one of the isothermal amplification strategies for biological analysis, a double specific nuclease (DSN)-based sensing system exhibits the advantages of simple principle, convenient operation, and high specificity [4,5,6,7]. DSN can specifically cleave DNA in DNA-DNA or DNA-RNA duplexes but shows no activity toward single-stranded DNA (ssDNA) or double-stranded RNA (dsRNA) [8,9]. The principle of DSN-assisted signal amplification is mainly based on target miRNA recycling. Thus, a few miRNAs could trigger the cleavage of considerable amounts of DNA in DNA-RNA heteroduplexes, leading to the release of a large number of signal molecules, including enzymes, electroactive molecules, fluorophores, nanoparticles, and nucleotide sequences [10,11,12,13,14,15,16,17,18,19,20,21,22,23,24]. The concentration of miRNAs can be determined by monitoring the optical or electrical signal change induced by the release of signal molecules. In these methods, signal-labeled capture probes are usually attached onto a solid surface and DSN-based enzymatic reactions happen at the solid–liquid interface. The heterogeneous sensing systems show the advantages of less sample consumption, ultrahigh sensitivity, and excellent selectivity. However, the probe-immobilized methods may cause several drawbacks. First, the steric hindrance effect of the solid surface may affect the configurational freedom of capture probes and prevent interactions between DNA-RNA hybrids and nucleases, thus decreasing the hybridization and cleavage efficiency [25,26,27,28,29]. Second, the high salt concentration on the probe-modified solid surface may limit the activity of nucleases [30,31]. In addition, the immobilization and removal of capture probes require laborious and time-consuming procedures. In contrast to heterogeneous sensing systems, homogeneous assays have the merits of a simple operation and high cleavage efficiency. Although a few DSN-based homogeneous methods have been developed and used for miRNA detection [32,33], they are usually involved in complex procedures, special instruments, and/or the use of additional enzymes or nanomaterials for signal amplification. Thus, it is of importance to develop a simple, sensitive, and high-throughput homogeneous method for the detection of miRNAs with DSN-assisted signal amplification.

Magnetic beads (MBs) have been extensively used for the development of various biosensors for detective applications. Magnetically assisted sensing systems show remarkable advantages in the separation and pre-concentration of targets and high throughput detection of multiple samples [34,35,36,37]. Streptavidin (SA) is a homotetramer protein that can bind up to four biotin molecules with a high affinity. Biotin is a commonly used reagent for labeling biomolecules due to its small size and highly selective and stable interaction with SA. Many SA-modified materials, including MB-SA, are commercially available for the collection and immobilization of biotin-labeled proteins or nucleic acids [38]. In this work, we proposed a homogeneous strategy for the detection of miRNAs with DSN-assisted signal amplification. In the absence of target miRNA, the biotinylated fluorescently labeled DNA probes were captured by MB-SA through fast and strong SA-biotin interactions, thus causing a decrease in the fluorescence. In the presence of target miRNA, the capture probes would be digested by DSN, and the released signal molecules in solution showed a high fluorescence. The reaction rate of DSN for the DNA capture probes immobilized on the MB surface and dispersed in solution was investigated. The proposed strategy can be used for the design of various immobilization-free miRNA biosensors by changing the type of signal molecules. Moreover, magnetically assisted sensing systems could be used for the high-throughput detection of multiple miRNAs with different fluorescently labeled sequences.

## 2. Materials and Methods

### 2.1. Chemicals and Reagents

DNA, miRNAs, and Trizol reagent were ordered from Sangon Biotech. Co., Ltd. (Shanghai, China). Their sequences are Biotin-TCAACATCAGTCTGATAAGCTA-FAM (Bio-DNA-FAM), UAGCUUAUCAGACUGAUGUUGA (miRNA-21), UAGCUUAUCGGACUGAUGUUGA (single-base mismatch), UUGCUUAUCGGACUGAUCUUGA (three-base mismatch), and GUAAGGCAUCUGACCGAAGGCA (non-complementary). DSN was obtained from Evrogen Joint Stock Company (Moscow, Russia). MB-SA was purchased from Thermo Fisher Scientific (Shanghai, China). Other reagents were of analytical grade and used without further purification. The miRNA samples were prepared freshly using RNase-free ultrapure water. The hybridization and reaction solutions were prepared with TNE buffer (pH 7.4). All aqueous solutions were prepared with ultrapure water treated by a Millipore system.

### 2.2. Procedures for miRNA Detection

In total, 150 μL of 500 nM DNA probe was mixed with 25 μL of miRNA sample in TNE buffer at 45 °C. After incubation for 10 min, 25 μL of 0.1 U DSN solution was added to the mixture. After reaction for a given time, 10 μL of 10 mg/mL MB-SA suspension was added to the mixed solution and incubated for about 5 min. Under a magnetic force, the supernatant solution was taken out for assays to be conducted. The signals were collected on a Cary Eclipse fluorescence spectrometer (Palo Alto, CA, USA) with an excitation wavelength of 492 nm.

### 2.3. Assays of miRNAs in Cell Lysates

The cell lysates were extracted from MCF-7 and Hela cells with the procedures detailed in previous reports [39,40]. Briefly, the cells were counted, collected, and washed with phosphate buffer. Then, Trizol reagent was added to extract total RNA according to the manufacturer’s protocol. The collected lysates were centrifuged at 10,000 rpm for 15 min and the aqueous supernatants were diluted 10-fold with TNE buffer. Then, 25 μL of the diluted supernatant with additional treatment was added to 150 μL of probe solution for quantification assay. Other procedures for the assays of miRNA in the lysates were the same as those of the standard samples.

## 3. Results and Discussion

### 3.1. Detection Principle

**Scheme 1:**
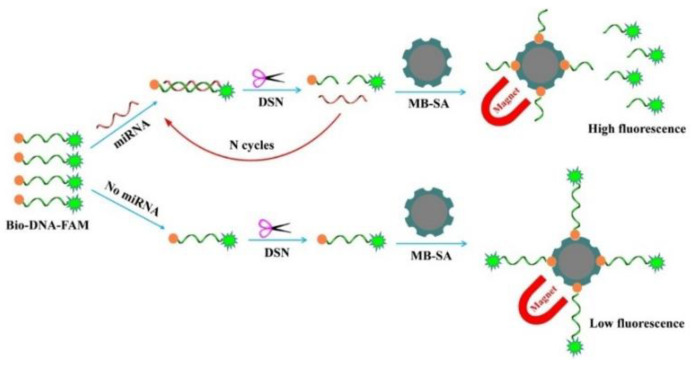
Schematic representation of the magnetically assisted immobilization-free method for miRNA detection.

The mechanism of the magnetically assisted detection of miRNA is depicted in Scheme 1. The DNA probe labeled with a biotin tag and a fluorophore at two ends (denoted as Bio-DNA-FAM) could hybridize with the target miRNA to form a DNA-miRNA heteroduplex. The DNA strand in the duplex was then selectively digested by DSN. The released miRNA could hybridize with another Bio-DNA-FAM probe and initiate the next cleavage event. In this way, the target miRNA-initiated enzymatic digestion of Bio-DNA-FAM probes by DSN was recycled, leading to the release of numerous FAM-labeled fragments. The released fragments could not be removed by MB-SA under the magnetic force and thus showed a strong fluorescence. In the absence of target miRNA, the Bio-DNA-FAM probe remained intact and would be removed by MB-SA through streptavidin–biotin interaction. In this case, the solution showed weak fluorescence. Therefore, the fluorescence intensity is proportional to the level of target miRNA. Through the signal amplification of DSN, miRNA at a low abundance would be detected. The method should be simple and sensitive since it does not require the pre-immobilization of DNA probe on a solid surface, thus improving the hybridization and cleavage efficiency without the effect of steric hindrance.

### 3.2. Feasibility of this Method

**Figure 1:**
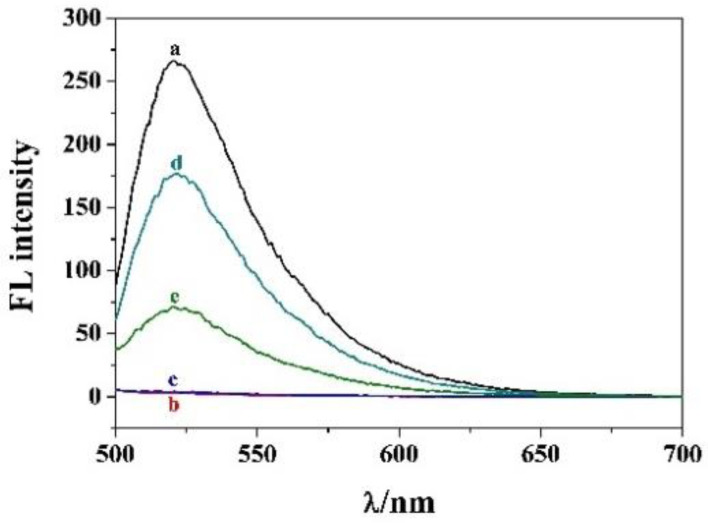
Fluorescence spectra of Bio-DNA-FAM before (curve a) and after (curve b) incubation with MB-SA, as well as the mixture of Bio-DNA-FAM/miRNA-21 (curve c) and Bio-DNA-FAM/miRNA-21/DSN (curve d) after treatment by MB-SA. Curve corresponds to that of incubating the MB-SA-Bio-DNA-FAM conjugates with the mixture of miRNA/DSN. The used concentrations of Bio-DNA-FAM, DSN, and miRNA-21 were 500 nM, 0.1 U, and 100 pM.

To probe the feasibility of the method, miRNA-21 was tested as a target model because its expression level is closely related to many cancers. As shown in Figure 1, the Bio-DNA-FAM probe emitted a strong fluorescence at 521 nm (curve a). When it was incubated with MB-SA under magnetic force (curve b), the fluorescence peak disappeared, indicating that the probe could be captured and removed by MB-SA. When the Bio-DNA-FAM probe was mixed with miRNA-21 in the absence of DSN, the fluorescence signal was close to the background value (curve c), indicating that the hybridization of miRNA-21 with Bio-DNA-FAM did not limit the capture of the probe by MB-SA. However, when the DNA-miRNA duplexes were incubated with DSN for a given time and then treated by MB-SA under the magnetic force, the fluorescence signal was intensified greatly (curve d). The result suggested that the Bio-DNA-FAM probes were enzymatically digested by DSN, thus releasing a large number of FAM-labeled fragments into the solution. To study the effect of steric hindrance on the hybridization/cleavage efficiency, the DNA-miRNA duplexes were pre-immobilized onto the MB-SA surface, and then DSN was added to the suspension for enzymatic digestion under the same reaction conditions. As a result, a smaller signal (curve e) was observed in contrast to the immobilization-free strategy (curve d). The result implied that the cleavage efficiency was reduced by the steric hindrance, which is consistent with that in the previous works [27,41]. Thus, our proposal could improve the detection sensitivity without increasing the operation complexity.

### 3.3. Optimization of Experimental Conditions

**Figure 2:**
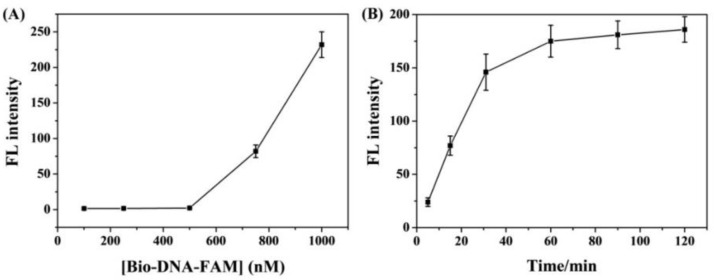
Effect of probe concentration (**A**) and incubation time (**B**) on fluorescence intensity. The concentrations of Bio-DNA-FAM, miRNA-21, and DSN were 500 nM, 100 pM, and 0.1 U, respectively.

To achieve optimum analytical performance, the experimental conditions, such as the probe concentration and cleavage time, were investigated. Because the background signal would be dependent upon the probe concentration, we first measured the fluorescence signals of different concentrations of Bio-DNA-FAM after treatment by MB-SA under a magnetic force. As shown in Figure 2A, when the probe concentration was lower than 500 nM, the fluorescence intensity was negligible. Over the value, the signal began to increase, indicating that the surface of MB-SA was saturated by the probe through the SA-biotin interaction. The abundance of miRNAs in biological samples is extremely low (usually in the range of femtomolar to picomolar), and the background signal can affect the sensitivity of the analytical method. Thus, a low concentration of probe was used in the following trials. When the incubation time for the mixture of DNA/miRNA-21 and DSN was increased, the fluorescence intensity was gradually intensified and began to level off beyond 60 min for 100 pM miRNA-21 (Figure 2B). To attain high sensitivity and save detection time, the samples were incubated for 60 min to achieve hybridization and cleavage recycling.

### 3.4. Sensitivity for miRNA-21 Detection

**Figure 3:**
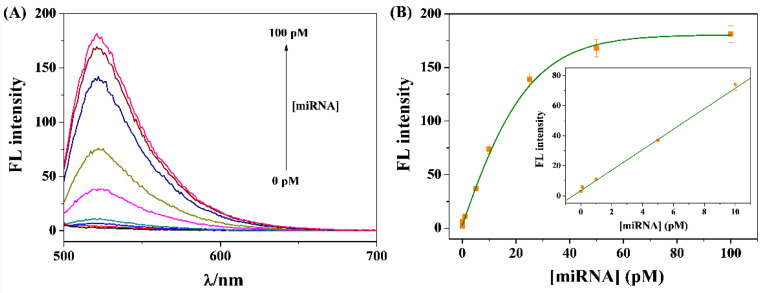
(**A**) Fluorescence spectra for the detection of different concentrations of miRNA-21 (from to bottom to top: 0, 0.01, 0.1, 1, 5, 10, 25, 50, and 100 pM). (**B**) Calibration curve between fluorescence intensity and miRNA-21 concentration. The errors were deduced from three replicate measurements. The inset shows the linear portion of the calibration curve.

Under the optimal conditions, various concentrations of miRNA-21 were determined to indicate the analytical performance. As shown in Figure 3A, the fluorescence signals were intensified with the increase in the miRNA concentration in the range of 0~100 pM. A good linear relationship was found at the concentration range of 0.01~10 pM (Figure 3B). The linear equation can be expressed as FL = 3.47 + 6.23 [miRNA-21] (pM). The detection limit as the minimum detectable concentration (0.01 pM) [42] is lower than that achieved by other DSN-based fluorescence methods with nanomaterials as the quenchers, including gold nanoparticles, graphene oxide, molybdenum disulfide, and polydopamine-coated magnetic nanoparticles (Table 1). The sensitivity was also comparable to that achieved by DSN plus other signal-amplified techniques [4,6]. The high sensitivity can be attributed to the immobilization-free strategy, the low background signal, and the high hybridization and cleavage efficiency.

### 3.5. Selectivity

**Figure 4:**
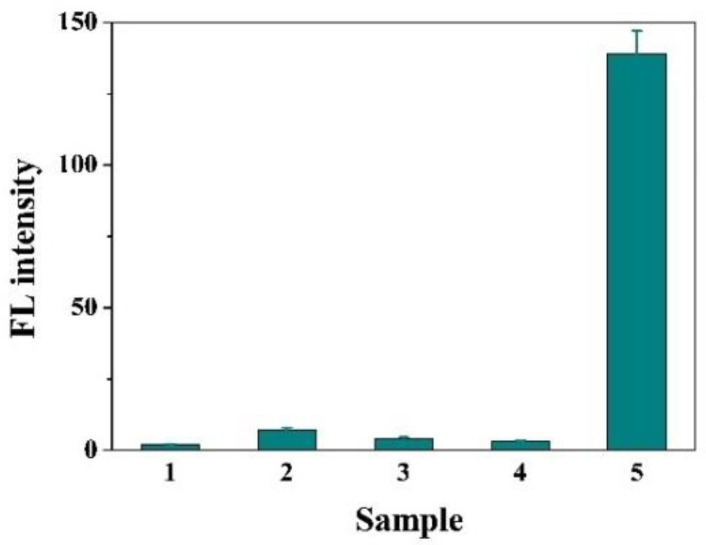
Selectivity of the method. Bar 1, buffer blank; bar 2, single-base mismatch; bar 3, three-base mismatch; bar 4, non-complementary; bar 5, miRNA-21.

To indicate the selectivity of the strategy, a series of miRNA strands were tested, including miRNA-21 and its base-mismatched and non-complementary sequences. As shown in Figure 4, only the target miRNA-21 caused a significant increase in the fluorescence signal. Although the single-based mismatched sequence may hybridize with the probe at room temperature, it did not induce an obvious enhancement in the fluorescence signal. The result should benefit from the high specificity of DSN and the high reaction temperature where the mismatched sequences could not form DNA-RNA duplexes. In addition, the probability and abundance are very low for the existence of the miRNA-21 variant with a single mismatch in the real sample. By labeling the DNA probes with different fluorophores, we believe that the immobilization-free method could be used to simultaneously determine multiple types of miRNAs with a simple operation procedure and high detection throughput.

### 3.6. Assays of miRNA-21 in Cellular Lysates

**Figure 5:**
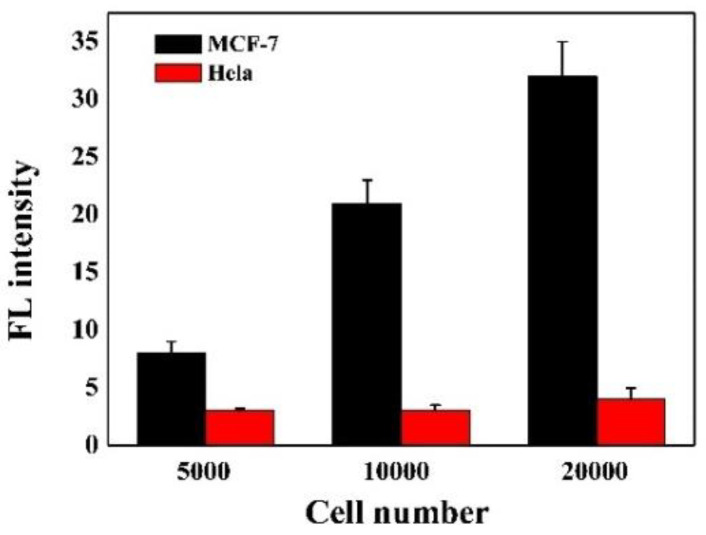
Results for the assays of miRNA-21 in lysates extracted from MCF-7 and Hela cells.

To evaluate the validity of our method for real sample assays, the levels of miRNA-21 extracted from two types of cancer cell lines were determined. As depicted in Figure 5, the signals increased significantly with an increasing concentration of MCF-7 cells, while no obvious change was found for Hela cells even at a concentration higher than 10,000 cells. This indicated that the expression level of miRNA-21 in MCF-7 is higher than that in Hela cells. The result is in agreement with that found by the heterogeneous biosensors [39,40,56,57], indicating that our method can be used to measure the expression levels of miRNA in biological samples. The detectable cell number is higher than that of the heterogeneous assays, which may be attributed to the differences in the used sample volume and the expression level as well as extraction method for miRNA-21. We believe that the sensitivity would be further improved by the signal amplification of enzymes or nanomaterials in combination with the use of a more sensitive fluorescence spectrometer.

## 4. Conclusions

In summary, an immobilization-free fluorescence method with DSN-assisted signal amplification was developed for the sensitive detection of miRNAs. The effect of steric hindrance on the hybridization/cleavage efficiency was also investigated. The detection limit of this method is lower than that of other DSN-based fluorescence assays and even comparable to that achieved by multiple signal amplification strategies. It was successfully employed to monitor the expression levels of miRNA-21 in two cancer cell lines. In addition, the strategy does not require the pre-immobilization of a DNA probe on a solid surface for hybridization/cleavage recycling, thus eliminating the effect of steric hindrance and improving the detection efficiency. We believe that the proposal shows great potential for the simultaneous detection of multiple miRNAs by matching the DNA probes with different fluorophores.

## Figures and Tables

**Table 1:** Analytical performances of DSN-based fluorescence methods for miRNA detection.

Materials/Reporters	Linear Range	Detection Limit	Ref.
AuNPs/dye	5–200 pM	5 pM	[41]
AuNPs/dye	0.1–1000 pM	33.4 fM	[43]
AuNPs/dye	0.1–10 pM	45 fM	[44]
PS@Au/dye	1–1000 pM	50 fM	[45]
MWCNT-AuNCs/dye	0.1–1000 pM	33.4 fM	[46]
GO/dye	0.5–1000 pM	160 fM	[47]
GO/dye	5–40 nM	132 pM	[48]
MoS_2_/dye	1–10,000 pM	10 fM	[49]
MoS_2_/dye	0.5–50 nM	426 pM	[50]
CCP/dye	10–200 pM	4.6 pM	[51]
PDA-PEI/dye	0.8–50 nM	0.52 nM	[52]
MNPs@PDA/dye	5–5000 pM	0.42 pM	[53]
MB/QDs	0.5–10 pM	0.5 pM	[54]
Cu_3_(PO_4_)_2_ NFs/dye	1–100,000 pM	0.23 pM	[55]
MB-SA/dye	0.01–10 pM	0.01 pM	this work

Abbreviations: AuNPs, gold nanoparticles; PS@Au, gold nanoparticle-coated polystyrene microbeads; MWCNT-AuNCs, multi-walled carbon nanotube–gold nanocomposites; GO, graphene oxide; MoS_2_, molybdenum disulfide; CCP, cationic conjugated polymer; PDA-PEI, dopamine and polyethyleneimine (PEI) copolymerized nanodots; MNPs@PDA, magnetic nanoparticles coated with polydopamine; MB, magnetic bead; QDs, quantum dots; Cu_3_(PO_4_)_2_ NFs, Cu_3_(PO_4_)_2_ hybrid nanoflowers.

## Data Availability

Not applicable.
